# Hospital and Institutional News

**Published:** 1918-06-01

**Authors:** 


### HOSPITAL AND INSTITUTIONAL NEWS.

### SIR ARTHUR SLOGGETT, K.C.B., K.C.M.G., K.C.V.O.

Lieut.-Gen. Sir Arthur Sloggett, having completed the period of his appointment as Director-General of Medical Services, and passed the age limit for service on the active list, has retired in accordance with the general principle that officers so situated shall be replaced by younger officers of proved ability and experience. General Sloggett received his first commission nearly forty years ago, and has served with distinction in the wars of the last twenty years. He has held the highest offices in his department in India and at home. He has had control in France since the outbreak of the war in 1914. The appointment was very grateful to the French people, with whom Sir Arthur Sloggett has been popular, whilst the high state of efficiency throughout under his direction has been one of the most notable and remarkable factors in the medical and hospital arrangements for the care and reception of the sick and injured. The entire organisation has in fact won the admiration and eloquent comment of many competent judges who have had full opportunities to study the arrangements and appreciate them at their true value. Amongst his many duties, the reception and entertainment of and provision for visitors from the Homeland, of all ranks, possessing many and varying characteristics, have exhibited Sir Arthur Sloggett in his element, and received the appreciative acknowledgments of all who have been privileged to enjoy his hospitality, and to become acquainted with his organisation and work.

### THE NEW D.G.M.S. IN FRANCE.

The Empire in general and the Medical Services in particular are to be congratulated on the appointment of Major-General C. H. Burtchaell, C.B., C.M.G., to succeed Lt.-Gen. Sir Arthur Sloggett. General Burtchaell has been Assistant Director-General in France during the whole of the present war. He has initiated much of the organisation which has revolutionised the transport and care of the sick and wounded. He has acquired the great advantages of an intimate knowledge and long experience of every detail of the administration and of continuous and intimate association with the staff officers of the various administrative departments, so that his new appointment affords every prospect of the undisturbed continuity of a policy which has produced such satisfactory results. Gen. Burtchaell is the third son of Peter Burtchaell, C.E., of County Kilkenny, Ireland; born in 1866; M.B., B.Ch., Trinity College, Dublin, 1889; appointed Surgeon R.A.M.C., 1891, Major, 1898, Lieutenant-Colonel, 1906, Colonel, 1915, and Major-General, 1917. He is an officer of very wide experience, having held the appointments of Staff Officer of Medical Services, 1st Division, during the South African War; Principal Medical Officer, South African Constabulary; Deputy Assistant Director of Medical Services in Ireland; and Assistant Director-General, War Office, from 1910 to the outbreak of war. He served in the N.W. Frontier of India campaign, the Tirah campaign (mentioned in dispatches, and promoted surgeon-major), and the South African War (mentioned in dispatches), being present at several actions, including Magersfontein. For his services in the present war he has been six times mentioned in dispatches and been awarded the C.B., C.M.G. and the Legion of Honour.

### THE NEW SECRETARY AT HARTSHILL.

Mr. Wm. Stevenson, the new House Governor and Secretary of the North Staffordshire Infirmary, Hartshill, whose appointment we announced in our issue of May 18, page 133, has been considerably boomed by the Buxton papers. Mr. Stevenson has served the Devonshire Hospital in various capacities for thirty-six years, becoming its secretary in 1903. The Devonshire Hospital and Buxton Bath Charity is of course a special hospital, and the Buxton papers claim that Mr. Stevenson has been the presiding genius and the controlling influence of the fate of this institution since he became its general superintendent as well as its secretary. The local papers declare that he has provided Buxton with the finest and most commodious hospital bathing installation for mineral water treatment in the country and probably in the world, which he had finished in 1915 and so providentially had ready for the soldiers crippled by rheumatism and kindred ailments in the trenches. Further, in 1916 he commenced his remarkable appeal to people all over the country for funds to wipe off the incubus of £13,000 of debt, which sum he raised in just over twelve months. Mr. Stevenson has filled many and varied offices. His remarkably fine bass voice made him a first-prize winner in the bass solo of the Buxton Musical Festival, a member of a prize-winning quartet and the male voice choir. He has arranged the choruses for successive conductors of the Gardens orchestra. He has filled notable places in the history of the Buxton Football Club. Mrs. Stevenson, the *Buxton Advertiser* states, has been, even more than her husband probably, connected with the musical side of Buxton social life, and has long delighted Buxtonians by her artistic and gifted vocalisation.

### A HOSPITAL CONSTITUTION RESTING ON COMPLETE DEMOCRACY.

We have had a recent opportunity of inspecting the North Staffordshire Infirmary, Hartshill, and seeing the remarkable and great changes which have been made throughout this important hospital. The atmosphere of the Hartshill Hospital to-day is splendid. Everybody is working together in the most cordial spirit of purposeful co-operation, imbued with the proper feeling that to-day their hospital entails upon them the privilege of giving continuously their very best work and utmost effort to make it as perfect throughout as possible. Dr. Rhodes has proved to be an untiring worker, whose one object has been to devote himself entirely to the work and to make the residents throughout the districts which this hospital serves realise that, **it is their hospital,** and that its success requires the co-operation and cordial continuous help of all classes, so as to secure that its constitution shall rest upon a complete democracy of the present governors and subscribers, who represent all types of the community. The management includes professional men, men of business, workers and independent residents, rich and poor, masters and men. Each of these sections sends its representatives as members of the various committees and of the Board of Management. It is their hospital, managed by themselves with an ability and purposeful intelligence that has saved the institution, and made it the instructive representative it is, of the voluntary hospital at its best, under modern conditions, resting upon a complete democracy. Each representative is rejoiced to give of his available time in personal service to the required extent, as and when their hospital needs such help. The opportunity to render this service is held to be a privilege, and properly valued accordingly.

### DEATH OF A FORMER HOSPITAL CHAIRMAN.

The death of Mr. Thomas Alexander Cook, who from 1901 to 1915 was chairman of the West Ham (now Queen Mary's) Hospital, removes an enthusiastic and able hospital worker, who contributed more than once to these columns, and many a time passed on hints or news of some successful piece of organisation which he thought would be helpful to others. During his chairmanship many additions were made to his institution, in which he took the most loving personal interest. Since the war he has been chairman of the local pensions committee. He served from the start on the committee for the purchase of Army camp refuse, of which he became chairman. The hospital grew and developed under his leadership, and its re-christening really marked a new stage in its scope and reputation. Mr. Cook was genial and easy to work with, and nothing seemed a trouble to him in connection with the philanthropic work which filled his leisure. A victim to pneumonia, he died at the comparatively early age of fifty-six, and will be missed by all who knew his qualities.

### SECRETARIES AND THE TRIBUNALS.

The new secretary of the Birmingham Children's Hospital, who is aged thirty-two, married, and has been passed Grade 2, applied to the Birmingham Local Tribunal for the renewal of his exemption from military service. The chairman of the hospital committee, Mr. David Davies, remarked that his official, who had previously been assistant secretary at the General Hospital, became secretary to the Children's Hospital in December last. He was the only man employed in administration by the committee, which was very anxious to retain his services, for substitutes were most difficult to find. The National Service representative urged that the applicant was only thirty-two, and that another hospital secretary, who was thirty-nine, had already been taken. Mr. Solomon Hill, the chairman, said that the need for men was too great to allow a man of thirty-two to be retained. The Tribunal granted three months' exemption, which would be final.

### FOR THE FOURTH TIME OF ASKING.

A curious document is the report and list of contributions to the "Our Day" collection in 1917, the third which was organised by the Joint War Committee of the British Red Cross Society and the Order of St. John. We are accustomed to huge totals in this war; they have ceased to excite more than a languid interest. But in this report one learns how the big total was made up, what contribution came from Persia, what from Paraguay, and how subscriptions were received from the Windward Islands. Exactly who will be interested enough to pay a shilling for the report, however, is not apparent. It is necessarily all acknowledgments, and there are eighty pages of them. "Our Day" 1918 will be held on Thursday, October 24, for the fourth time.

### "A FREE HOSPITAL SERVICE."

Mr. R. S. Dawson, the chairman of the Bradford Board of Guardians, has suggested a reconstructed hospital service. The City Council, he suggests, should form a hospital authority to work with the health committee. The authority should be composed of councillors, doctors, hospital representatives, and Guardians. All the hospitals and institutions of the city, whether rate-supported or not, should be controlled by this authority, which would justify its control of voluntary institutions by financial grants. Part of the scheme is to amend the Insurance Act so as to secure a free hospital service for all insured persons and their families. Mr. Dawson believes that overlapping would be prevented by his scheme, the kernel of which is the amendment of the Insurance Act. Such schemes, however, mean little until their details are placed before us, though a Guardian's view is of interest.

### WOMEN HOSPITAL SECRETARIES.

The new secretary of Keighley Victoria Hospital is Miss Hoyle, who has been appointed out of twenty-eight candidates to succeed Mrs. Grace. Miss Hoyle, who has been a headmistress of a girls' school in London and engaged in teaching till 1901, was the only woman co-opted on the new Education Authority at Keighley on the extinction of the old School Board.

### WILLIAM DUDLEY'S CHARITY.

A little-known institution has been brought into the light as an accident of the recent appeal on behalf of the Birmingham Children's Hospital. This is "William Dudley's Charity," which, according to a supporter of the hospital, was founded nearly fifty years ago with the gift of £100,000 by William Dudley. It had apparently three aims to serve—to make small loans to impecunious Birmingham tradesmen of good character; to make grants to old and impecunious Birmingham tradesmen; and "to distribute the surplus income amongst such Birmingham institutions for the relief of human suffering as shall appear to the trustees most deserving of help." The founder directed that the City Council should elect four representative members to form the committee of management. It is asserted that the first object alone has been freely advertised, and, presumably, fulfilled, and that the committee publishes no report or annual statement of accounts. The City Council, as the body which elects the committee of the fund, is therefore invited to do so. Such a statement would reveal the amount of surplus income available for distribution among deserving institutions. These facts would be worth knowing, and local hospitals have sufficient interest in them to ensure that they will be revealed.

### A WILL AND THE WAR.

The Stratton Cottage Hospital, Devonshire, has been placed in a difficulty because a bequest of £1,500 was left to it on condition that the additional ward which this was intended to provide should be built within two years of the death of the testator. The decision of the Minister of Munitions on a request for permission to build is awaited by the committee and the secretary, Mr. J. Martyn, who recently succeeded Mr. F. Carver in this post. We hope either that the Ministry will give the necessary permission, or that the validity of the bequest will not be imperilled by a refusal for which the hospital is not responsible.

### THE MARRIED ASSISTANT MEDICAL OFFICER.

Bermondsey Board of Guardians have regretfully declined to grant an increase of salary to Dr. W. E. Reid, assistant medical officer. He receives £500 a year, and Dr. E. S. Bell, the chief medical officer, in supporting his application, pointed out that Dr. Reid had to pay house rent, heavy rates and taxes, and to provide a servant, since he had children. Out of his salary he was unable to live at the present, and he informed Dr. Bell that he had to draw upon savings. Dr. Bell rightly pointed out that this salary of £500 a year was less by a considerable amount than that of a resident *locum tenens* at the minimum salary of seven guineas a week in addition to all allowances, and, if Dr. Reid resigned, he doubted whether a suitable successor could be obtained. Nevertheless, the Guardians declined. The effect of their action is to penalise married medical officers.

### PERMITS FOR SURGICAL INSTRUMENTS.

It is now necessary to obtain a "repairs and maintenance permit" from the Ministry of Munitions of War for supplies of forceps, clamps, and other metal surgical instruments. When supplies are required a priority certificate (these are obtainable at any post office) must be filled up in duplicate, and the number of the hospital's permit quoted on this. The certificate is forwarded to the instrument-maker and the duplicate retained. These are filed, and a return of all purchases made under the authority of the permit is required by the Priority Department every six months. This permit does not authorise the purchase of platinum or aluminium or copper articles for cooking or heating. Hospitals who have reduced clerical staffs will probably find it advantageous to anticipate their requirements and order surgical instruments less frequently, so that the number of duplicates will be kept as low as possible, and so reduce the amount of bookkeeping.

### "COLIS" FOR CAPTURED PRINTERS.

The French printing craft set up in 1915 a fund on behalf of printers of all grades, employers and employed, of any Allied country, who have become prisoners of war or to whom the German invasion has brought distress. Since that time funds are collected with more difficulty, while the rations given to the prisoners and the provision by German authorities of clothes and needful things seem steadily to have become worse. Therefore efforts which the craft in France has been exerting on behalf of its distressed members have also been assisted by printers of the United States; and to-day the French craft invites the aid of the printing and kindred trades in the United Kingdom. The invaded district includes such towns as Lille, Arras, Roubaix and St. Quentin, which, with their environs, have a population of, roughly, a million people. In this essentially industrial region printing establishments were numerous and important, and great numbers of printing works have been despoiled and printers, employing and employed, have found themselves without resources. For the succour and cheer of printers in distress, French or Belgian, or of any Allied nation, a war service bureau of the printing craft, called "Œuvre de Guerre des Industries du Livre," was established in 1915, under the auspices of the French Master Printers' Association. Continuous collections from the French printing craft have been made by this "Œuvre de Guerre des Industries du Livre," and the donations have been published monthly in the official Journal of the Association. The work, it may be mentioned, is associated with the Red Cross organisation in France. So far as concerns prisoners of war, a large part of the assistance is in the form of eagerly welcomed "colis," *i.e.*, parcels of food and clothing. A small committee has been formed, under the auspices of the Master Printers' Federation and the Institute of Printers and Kindred Trades of the British Empire, to raise a fund worthy of the British craft in support of this "Œuvre de Guerre" collection. Mr. J. H. Williams, of Messrs. Williams, Lea and Co., Ltd., is chairman of that committee. Mr. R. A. Austen-Leigh, of Messrs. Spottiswoode, Ballantyne and Co., is hon. treasurer, and has opened an account at the bank of Messrs. Hoare, 37 Fleet Street, E.C. 4. Mr. Frank Colebrook, of 146 Fleet Street, E.C. 4, is acting as hon. secretary. This committee appeals for help for these fellow-craftsmen, whether distressed through the ruin of establishments in which they worked, or whether prisoners of war. The French fund has succoured 3,000 of them, but there are 7,000 more who need help. This appeal is submitted with confidence to printers of Great Britain, who, though they have undoubtedly had their own serious difficulties since war broke out, have escaped the horrors of invasion. Hospital men, who owe so much to the printing trade, will welcome this opportunity of helping.

### WOMEN AND THE HOUSING PROBLEM.

At a recently held conference on the housing question in Edinburgh, one woman speaker suggested that the cheap new houses it is hoped to erect " after the war " should be designed chiefly by women, because they had learned by practical and often bitter experience what was needed. Another proposed that every house should have a room where the housekeeper could have some moments of privacy and solitude! Though we have advanced since the days of universal basements, a single source of water supply, and four or five flights of tortuous stairs, modern architects would be well advised to seek the counsel of the woman when designing the houses in which their work is done. The kitchen, scullery, and larder of the usual suburban villa are as mean and cramped as commercial stinginess can make them. Since, however, they are usually " run up " by a speculative builder, the architects can hardly be blamed. The only remedy seems to be the disagreeable one of enforcing more stringent bye-laws in respect of the plans which local authorities will approve.

**URGENT NOTICE.**

To ensure a copy of The Hospital regularly, readers must place a definite order with their Newsagent TO-DAY. After the middle of June, in consequence of the new Paper Restrictions Order, it will not be possible for Newsagents to stock copies for chance customers.

DO NOT DELAY. ORDER

**"THE HOSPITAL" TO-DAY**

and obtain your copy regularly EVERY THURSDAY.

### THE FIRST PUBLIC WOMAN DENTIST.

The claim that the Essex Education Committee was the first public body to appoint a woman as school dentist has been successfully challenged by the Secretary for Education of the Dorset County Council. The Dorset Education Committee, it appears, appointed a woman as school dentist in 1913, and found her so valuable that when she married another woman was appointed with equal success. After two years the second woman also left to take up another public appointment. The woman dentist, therefore, is coming to stay. So far she has not invaded private practice. When she does, will she adopt the modern fashion of having an assistant always with her as those dentists do who have a woman in a nurses' uniform buzzing round the luckless patient? We asked lately a distinguished dental surgeon of the old school what he thought of this fashion. He replied with good-humoured contempt that it was part of a parade he had no use for, that he could attend to his patients himself except when anæsthesia was administered, and that if the notion of the nurse was to protect the dentist from hysterical patients, his experience had not found him so helpless as these juniors, or Americans, appeared to be.

### A TROPHY OF CAPTIVITY.

The recent arrivals of prisoners of war from Germany are mentioned in the May number of *The British Prisoner of War*. One of the trophies of captivity which was brought home was " a large chunk of prisoner's bread, which appeared to be full of chopped straw." News from the men of Ruhleben camp is now obtainable in the pleasant form of an exhibition of their handiwork, which the journal is preparing at the request of the Prisoner of War Department in Downing Street. The exhibits, which range from rat-skin objects to oil paintings, are due to arrive at the end of May. The exhibition of drawings by Mr. Wiggin, who returned from Germany last January after three years' imprisonment, at the Carfax Gallery, Bury Street, St. James', closed on May 11. A full account is given of the bureau which was started in the autumn of 1916 at Copenhagen to supply British prisoners in Germany with bread. The first of these bureaus was that opened at Berne in 1915 by Lady Grant Duff. An account of this is promised, but has been delayed by the uncertainty of the posts. A contribution again appears from *The Barb*, the camp magazine published at Treves, which seems to be the most lively of these journals.

### THIS WEEK'S DRUG MARKET.

The reaction from the quiet period through which the market has been passing has not set in as yet. A good deal of buying on account of the Medical Services is, however, going on; but private business is decidedly slow, and price changes of importance have not been numerous. Sugar-of-milk is much inquired for, but supplies are slow in arriving, and the consequence is that any buyer who is in immediate need of the article has to pay very dearly for it. The steady demand for American saccharin continues, and the value is well maintained; it is hoped that the output of British saccharin will be very substantially increased before long. A small business is being done in quinine at practically unchanged rates; the recent considerable arrivals have not materially affected the market. Both acetanilide and hexamine continue scarce with upward price tendencies. There is still a good demand for citric acid, and the value is firmly maintained. Chloral hydrate is scarcer and dearer. English refiners have again advanced their prices, but they are not freely offering supplies. There has been a considerable rise in the price of santonin. Carbolic acid is rather more freely obtainable at unchanged rates. Very little beechwood creosote is offered. So far as can be foreseen the crop of belladonna—both root and leaf—will be up to the average; the area under cultivation has been much increased.

